# Retraction: *Strychnos pseudoquina* modulates the morphological reorganization of the scar tissue of second intention cutaneous wounds in rats

**DOI:** 10.1371/journal.pone.0333454

**Published:** 2025-09-30

**Authors:** The PLOS One Editors

After this article [1] was published, concerns were raised regarding results presented in Figs 1-3.

Specifically:

- The following panels appear similar:
  - Fig 3 SAL Day 7 (F1) in [1] and Fig 1 Control Day 4 in [2]
  - Fig 3 OV Day 7 (F1) in [1], Fig 3 OV Day 14 (F2) in [1], and Fig 1 Balsam Day 4 in [2]
  - Fig 3 SS Day 21 (F3) and Fig 3 LE10 Day 0 (F0)
- The following panels appear to fully or partially overlap:
  - Fig 3 SAL Day 0 (F0) and Fig 3 OV Day 0 (F0)
  - Fig 3 LE5 Day 7 (F1) in [1], Fig 3 LE10 Day 7 (F1) in [1], Fig 1 Sunflower Day 4 in [2], Fig 1 Ointment Day 4 in [2]
  - Fig 3 LE5 Day 21 (F3) and Fig 3 LE10 Day 21 (F3)
- Fig 2 appears to show the quantification results of Fig 3.
- Figs 1B and 1C in [1] appear similar to Figs 1E and 1F in [3, corrected in 4] respectively.

Co-first author MMS stated that the results in Fig 3 are incorrect and that the original image data underlying Figs 1B and 1C are no longer available. They provided the original underlying data for Fig 3 as well as the individual-level quantitative data underlying the remainder of the figures in [1]. Upon editorial review, these underlying data were insufficient to resolve the above concerns, and raised further concerns for the reliability and validity of the published results.

In light of the above concerns, the *PLOS One* Editors retract this article.

RVG did not agree with the retraction. MMS did not respond to the final editorial decision. LLM, LSA, RDN, VVZ, and JPVL did not respond directly or could not be reached.

In addition to the concerns above, it was noted that the Materials and Methods section does not describe whether the rats were euthanized under anesthesia. During editorial follow-up, co-first author MMS stated that the animals were euthanized by cervical dislocation after intraperitoneal anesthesia with ketamine (180 mg/kg) and xylazine (30 mg/kg).

The Fig 3 SAL Day 7 (F1), OV Day 7 (F1), OV Day 14, LE5 Day 7 (F1), and LE10 Day 7 (F1) panels in [1] appear similar to content previously published in [2] under a CC BY license. These panels are subject to the license that applies to the original article [2].
